# Genome-wide identification of FoxO-dependent gene networks in skeletal muscle during C26 cancer cachexia

**DOI:** 10.1186/1471-2407-14-997

**Published:** 2014-12-24

**Authors:** Sarah M Judge, Chia-Ling Wu, Adam W Beharry, Brandon M Roberts, Leonardo F Ferreira, Susan C Kandarian, Andrew R Judge

**Affiliations:** Department of Physical Therapy, University of Florida, 1225 Center Drive, HPNP Building 1142, Gainesville, Florida USA; Department of Health Sciences, Boston University, Boston, Massachusetts USA; Department of Applied Physiology and Kinesiology, University of Florida, Gainesville, Florida USA

**Keywords:** Muscle Atrophy, Microarray, Proteasome, Cebpb, Fos, Transcription factors, Z-disc, Extracellular matrix

## Abstract

**Background:**

Evidence from cachectic cancer patients and animal models of cancer cachexia supports the involvement of Forkhead box O (FoxO) transcription factors in driving cancer-induced skeletal muscle wasting. However, the genome-wide gene networks and associated biological processes regulated by FoxO during cancer cachexia are unknown. We hypothesize that FoxO is a central upstream regulator of diverse gene networks in skeletal muscle during cancer that may act coordinately to promote the wasting phenotype.

**Methods:**

To inhibit endogenous FoxO DNA-binding, we transduced limb and diaphragm muscles of mice with AAV9 containing the cDNA for a dominant negative (d.n.) FoxO protein (or GFP control). The d.n.FoxO construct consists of only the FoxO3a DNA-binding domain that is highly homologous to that of FoxO1 and FoxO4, and which outcompetes and blocks endogenous FoxO DNA binding. Mice were subsequently inoculated with Colon-26 (C26) cells and muscles harvested 26 days later.

**Results:**

Blocking FoxO prevented C26-induced muscle fiber atrophy of both locomotor muscles and the diaphragm and significantly spared force deficits. This sparing of muscle size and function was associated with the differential regulation of 543 transcripts (out of 2,093) which changed in response to C26. Bioinformatics analysis of upregulated gene transcripts that required FoxO revealed enrichment of the proteasome, AP-1 and IL-6 pathways, and included several atrophy-related transcription factors, including *Stat3, Fos*, and *Cebpb*. FoxO was also necessary for the cancer-induced downregulation of several gene transcripts that were enriched for extracellular matrix and sarcomere protein-encoding genes. We validated these findings in limb muscles and the diaphragm through qRT-PCR, and further demonstrate that FoxO1 and/or FoxO3a are sufficient to increase *Stat3, Fos, Cebpb*, and the C/EBPβ target gene, *Ubr2*. Analysis of the *Cebpb* proximal promoter revealed two *bona fide* FoxO binding elements, which we further establish are necessary for *Cebpb* promoter activation in response to IL-6, a predominant cytokine in the C26 cancer model.

**Conclusions:**

These findings provide new evidence that FoxO-dependent transcription is a central node controlling diverse gene networks in skeletal muscle during cancer cachexia, and identifies novel candidate genes and networks for further investigation as causative factors in cancer-induced wasting.

**Electronic supplementary material:**

The online version of this article (doi:10.1186/1471-2407-14-997) contains supplementary material, which is available to authorized users.

## Background

Cachexia is a devastating condition that affects up to 80% of patients with cancer, particularly those with cancers of the lung and upper GI tract [1]. The condition is characterized by progressive weight loss due to significant skeletal muscle wasting, in the presence or absence of adipose tissue wasting. Importantly, the muscle wasting causes significant muscle weakness that negatively affects physical function and independence, and thus quality of life. In addition, muscle and body wasting during cancer is also associated with a reduced tolerance to chemotherapy [2], increased complications from surgical and radiotherapeutic treatments [3], higher rate of metastatic disease and decreased survival [4]. Therefore, developing treatment strategies to deter cancer cachexia is critically important to enhancing the quality of life and survival of cancer patients. However, in order for this to happen, a better understanding of the mechanisms which drive muscle wasting during cancer is needed.

Skeletal muscle wasting during cancer displays marked similarities to other atrophy-inducing conditions, in that the loss of muscle mass is characterized by increased protein degradation and decreased protein synthesis [1, 5]. However, recent studies demonstrate that muscle wasting during cancer is also related to disruptions in the dystrophin glycoprotein complex and muscle fiber integrity and impaired myogenic capacity [6, 7], thus emphasizing the unique and complex nature of cancer cachexia. The upstream molecules implicated in driving these muscle pathologies during cancer include several pro-inflammatory cytokines that are increased in the circulation of cancer patients and tumor-bearing mice [1, 8]. Intrinsic to the muscle, mechanistic evidence demonstrates the requirement of inhibitor of kappa B kinase beta (IKKβ) activation and the subsequent degradation of the inhibitor of kappa B alpha (IκBα) [7, 9, 10] for cancer-induced muscle atrophy and myogenic impairment. Also required for the atrophy phenotype during cancer are the transcription factors, Signal Transducer and Activator of Transcription 3 (STAT3), which acts downstream of IL-6 [11, 12], CCAAT/enhancer-binding protein beta (C/EBPβ), which is activated by p38 mitogen activated protein kinase (MAPK) [13], and activator protein-1 (AP-1) [14], which is activated through extracellular regulated kinase 1 and 2 (ERK 1/2) MAPK [15]. Lastly, evidence from our lab and another demonstrates that activation of Forkhead BoxO (FoxO) transcription factors also plays a causative role in cancer-induced muscle wasting [16, 17].

Skeletal muscle expresses three FoxO family members, including FoxO1, FoxO3 and FoxO4, with both FoxO1 and FoxO3a significantly upregulated in cachectic muscles from LLC and C26 tumor-bearing mice [9, 16]. Moreover, FoxO1 is also upregulated in skeletal muscle of human cancer patients, and was recently identified as a cachexia-associated gene [18]. Importantly, activation of the FoxO transcription factors is both sufficient to cause muscle atrophy and necessary for muscle wasting in response to numerous catabolic conditions, including cancer cachexia associated with Lewis Lung Carcinoma [16] and Sarcoma-180 [17]. Thus, these findings from both human cancer patients and animal models of cancer cachexia strongly support the involvement of FoxO in driving the muscle atrophy process. Despite this, the genome-wide gene networks regulated by FoxO during cancer cachexia are unknown. Indeed, although the FoxO factors are well established to regulate genes involved in skeletal muscle proteolysis through the ubiquitin proteasome pathway and autophagy, due to the complex nature of cancer cachexia, we hypothesize that FoxO regulates additional gene networks which promote the wasting phenotype. Indeed, identifying the broader gene targets regulated by FoxO is an important next step which may unveil novel insight into the mechanisms which promote cancer-induced muscle wasting.

The purpose of the current study was to determine the requirement of FoxO for locomotor muscle and diaphragm muscle wasting and weakness in response to Colon-26 (C26) adenocarcinoma, and provide the first genome-wide analysis of the genes and biological networks targeted by FoxO in response to C26 tumor burden. We found that FoxO is necessary for C26-induced muscle wasting of both locomotor muscles and the diaphragm, and that this was associated with its regulation of genes involved in not only proteolysis, but additional atrophy-related transcriptional pathways, including the IL-6 and AP-1 pathways. In addition we also identified FoxO as a novel regulator of gene repression during cancer cachexia, with the most enriched gene networks related to the structure and functional integrity of the extracellular matrix and muscle sarcomere. The data presented in this study thus highlights novel candidate genes and biological networks that are regulated downstream of FoxO that may be further explored as causative factors in cancer-induced muscle wasting.

## Methods

### Animals

Male CD2F1 mice weighing ~20 g were purchased from Charles River Laboratories (Wilmington, Massachusetts) and used for all animal experiments. Mice were maintained in a temperature and humidity-controlled facility with a 12-h light/dark cycle and water and standard diet were provided *ad libitum*. The University of Florida Institutional Animal Care and Use Committee approved all animal procedures.

### AAV vectors

**Figure 1:**
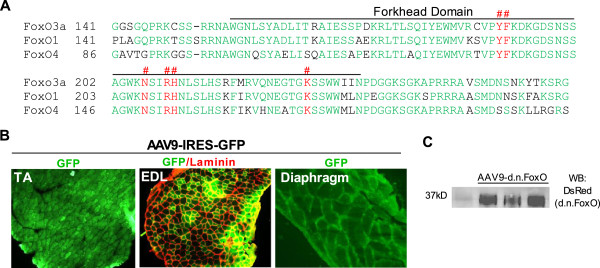
**Transduction of locomotor muscles and the diaphragm with AAV9-d.n.FoxO. (A)** Alignment of the dominant negative (d.n.) FoxO protein sequence, which includes amino acids 141–265 of mouse FoxO3a, with the corresponding mouse FoxO1 and FoxO4 amino acid sequences. FoxO1 shares ~85% amino acid sequence identity and FoxO4 75% sequence identity with the d.n.FoxO protein (shared amino acids denoted in green), all of which share >90% sequence conservation within this region. The 6 amino acid residues involved in DNA binding of the Forkhead Domain are highlighted in red and are denoted by hash marks (#) above the aligned sequences. **(B** **and** **C)** The AAV9 vectors driving expression of d.n.FoxO (or empty vector), which also contain an IRES driving the expression of GFP, were injected directly into the anterior hind limb compartment of mice to transduce the TA and EDL muscles, or injected directly into the intrathoracic cavity of mice to transduce the diaphragm. **(B)** Representative muscle cross-sections showing AAV9 transfection efficiency in the TA, EDL and diaphragm ~26 days post-injection as visualized via direct GFP fluorescence. **(C)** Confirmation that the d.n.FoxO protein was also expressed in muscles transduced with AAV9-d.n.FoxO was confirmed through western blot using an antibody against DsRed, which is fused to the d.n.FoxO protein.

The d.n.FoxO construct used to inhibit FoxO-dependent transcription encodes for amino acids 141–266 of human FoxO3a which encompasses only the FoxO3a DNA binding domain. The amino acid sequence of the d.n.FoxO protein shares 100% sequence identity with mouse FoxO3a (aa140-265), 85% sequence identity with mouse FoxO1 and 75% sequence identity with mouse FoxO4 (Figure 1), all of which share >90% sequence conservation within this region. Since the d.n.FoxO protein lacks a transactivation domain, the d.n.FoxO blocks DNA-binding dependent transcription by the FoxO factors through outcompeting endogenous FoxO factors for binding to FoxO DNA binding elements (FBEs) in gene regulatory regions. The d.n.FoxO is fused to a DsRed protein tag to allow for quantitation of the ectopic protein and has been used and described by our lab previously [16, 19]. The d.n.FoxO cDNA was sub-cloned into the SpeI and ClaI sites of pTR-UF12 under the control of a cytomegalovirus and chicken β-actin hybrid promoter. The pTR-UF12 shuttle vector also contains the internal ribosome entry site (IRES) that allows for the bi-cistronic expression of GFP for measurement of AAV9 transduction efficiency. The pTR-UF12-d.n.FoxO and pTR-UF12 (empty vector, ev) were packaged in rAAV9 and titered at the University of Florida Powell Gene Therapy Center Vector Core Laboratory using previously published methods [20]. The vectors were purified by iodixanol gradient centrifugation and anion-exchange chromatography, as described previously [20] and final formulations of AAV9-d.n.FoxO and AAV9-ev were provided in lactated Ringer’s solution.

### *In vivo*AAV delivery

Mice were acutely anesthetized with isoflurane gas (5%, induction; 3% maintenance) delivered via a nose cone. For AAV delivery to the TA and EDL, a small incision was made on the lateral side of the lower leg and the TA muscle exposed. Each vector was diluted in lactated Ringer solution such that 1 × 10^11^ vector genomes (VG) were injected in 25 μl along the tibia into the TA and EDL muscles, as previously described by others [21]. For targeting to the diaphragm, we performed a single intrathoracic injection of 1 × 10^11^ vector genomes/mouse in 400 μl of sterile lactate Ringer’s solution. This minimally invasive technique causes high and widespread transduction of the diaphragm [22, 23].

### Plasmid DNA vectors

Expression plasmids for the FoxO1 triple phosphorylation mutant (Addgene Plasmid 17547, deposited by Dr. Domenico Accili) and the FoxO3a triple phosphorylation mutant (Addgene Plasmid 10711, deposited by Dr. William Sellers ), have been described previously [24] and were injected and electroporated into mouse TA muscles as described by us previously [16]. The pGL4.20 luciferase reporter plasmids containing either a wildtype Cebpb promoter fragment (−516 to −1) or a mutated version which is mutated at both FoxO binding elements (FBE1 (−234 to −225) and FBE2 (−208 to −199)) were generous gifts from Dr. Akiyoshi Fukamizu, and have been described previously [25]. The pRL-TK-Renilla luciferase reporter plasmid was purchased from Promega (Madison, WI).

### Cancer cachexia

Murine C26 cells were obtained from the National Cancer Institute Tumor Repository (Frederick, MD, USA) and cultured as described previously [26, 27]. Cancer cachexia was induced in mice by injecting 5×10^5^ C26 cells (or 1× PBS as control) subcutaneously into each flank on the same day as AAV delivery. Muscles were harvested when the largest tumor diameter reached 1.5 cm (~26 days post-inoculation) when mice had lost ~15% of tumor-free body weight.

### Histochemistry

Transduction efficiency of AAV9 was determined in 10 μm cross-sections via direct visualization of GFP fluorescence using a Leica DM5000B microscope (Leica Microsystems, Wetzlar, Germany) prior to and/or following fixation with 4% paraformaldehyde and labeling of muscle fiber borders with Alexa Fluor-conjugated wheat germ agglutinin (Invitrogen) for 1 hr. Leica application suite, version 3.5.0 software was used to trace and measure fiber CSA as described previously [27].

### *In vitro*muscle contractile properties

The solutions and methods used for measurements of muscle isometric function in EDL muscles were described in detail previously [27, 28].

### C2C12 cell culture and IL-6 treatment

Mouse C2C12 skeletal myoblasts were purchased from American Type Culture Collection (Manassas, VA), and were cultured and transfected with plasmids as described previously by our lab [29]. Myotubes were treated on day 3 of differentiation with 10 ng/mL of IL-6 for either 0 or 3 hours prior to harvest and firefly/renilla luciferase activity was measured as previously described [29].

### RNA isolation

RNA was extracted from TA and diaphragm muscles using TRIzol as previously described [16]. Isolated total RNA was subsequently purified using an RNeasy Mini kit (Qiagen, Valencia, CA), according to manufacturer’s instructions. The resulting quantity and purity of total RNA was tested through absorbance spectrophotometry at 230, 260 and 280 nm, and the quality of RNA was tested on a 1% denaturing agarose gel. Synthesis of cDNA and qRT-PCR analyses from RNA isolated from the TA and diaphragm were performed as described previously [28] using a 7300 real-time PCR system and the following primers from Applied Biosystems (Austin, TX): Fbxo30 (NM_027968.3), Fbxo31 (NM_133765.4), Bach2 (NM_001109661.1), Socs3 (NM_007707.3), Ubr2 (NM_146078.3), Psma2 (NM_008944.2), Ubqln1 (NM_026842.4), Fos (NM_010234.2), Cebpb (NM_009883.3), Stat3 (NM_011486.4), Col6a2 (NM_146007.2), Myoz3 (NM_133363.3), atrogin-1/MAFbx/Fbxo32 (NM_026346.2), MuRF1/Trim63 (NM_001039048.2), Bcl3 (NM_033601.3), and Maff (NM_010755.3).

### Microarray

For microarray analysis, 16 total RNA samples from two conditions (control and tumor bearing) transduced with either AAV9-ev or AAV9-d.n.FoxO (4 samples per group, 4 groups) were sent to the Boston University Medical Center Microarray Core Facility for amplification, labeling, and hybridization on the mouse Affymetrix Gene 1.0 ST array (Santa Clara, CA, USA). This microarray is designed to measure the expression of 28,132 well-annotated genes. A total of sixteen array images were acquired by GeneChip Scanner 3000 TG and the image (expression) quality was assessed by the Affymetrix Expression Console (Santa Clara, CA, USA). The Expression File Creator module of the GenePattern platform was used to generate gene expression signal values [30] and were normalized by robust multi-array analysis algorithm (RMA) [31]. Brainarray MoGene 1.0 ST custom Chip Definition File v.16 was used for probe annotation [32]. The resulting expression data for 21,225 genes were uploaded for Principle Component Analysis (PCA) on the Genepattern platform [30]. We found one outlier from the AAV9-ev C26 group using PCA and thus, this sample was removed from further analysis. The expression values were log2-transformed and preprocessed by the Pre-ProcessDataSet module of GenePattern to include genes with expression values between 1-2^20^, a *min.fold.change* ≥ 2 and a *min delta* ≥ 1.2. The last two variation filters were set to eliminate genes that showed no expression change among the 15 samples but to include genes showing changes at low expression values. In the Pre-ProcessDataSet module, *min.fold.change* is defined as the fold change of the 2nd highest expression value among the 15 samples compared to the 2nd lowest value among the 15 samples, whereas *min delta* is defined as the difference between the 2nd highest expression value and the 2nd lowest value among the 15 samples [30]. Both *.cel* files and expression values were deposited into MIAME compliant NCBI Gene Expression Omnibus [33] with accession #GSE56555. Following these filtering and preprocessing steps, 20,432 genes remained.

Differential gene expression analyses were subsequently performed using the Comparative Marker Selection module in GenePattern [30], which compares mean differences between two groups by two-way parametric t-tests. To identify differentially expressed genes in muscles from tumor-bearing mice, expression values from the AAV9-ev control group were compared to the AAV9-ev C-26 group (using q ≤ 0.01 and −1.5 ≥ fold change ≥1.5-fold), which identified 2,194 genes. Then, to identify the direct or indirect FoxO target genes during cancer, the differentially expressed genes due to cancer were compared to expression values from the AAV9-d.n.FoxO C-26 group (q ≤ 0.01 and −1.5 ≥ fold change ≥1.5-fold), which identified 544 genes. Genes which were also significantly changed by AAV9-d.n.FoxO during control conditions (AAV9-ev control vs. AAV9-d.n.FoxO control, q < 0.01), were eliminated as FoxO target genes in response to the C26 tumor.

Upregulated or downregulated FoxO target genes in response to the C26 tumor were analyzed separately for their associated functional annotations using the DAVID Bioinformatics database [34]. Enriched terms and biological networks were identified using pre-selected default annotation categories, an EASE score (a modified Fisher Exact P-value) of less than 0.05 and an enrichment score greater than 1.5. Enriched terms were clustered using the Functional Annotation Clustering tool, which groups analogous annotations together to reduce redundancy in the report. FoxO target genes were also analyzed using the Broad Institute’s Molecular Signatures Database [35] to identify enriched canonical pathways and to identify the most commonly shared transcription factor binding motifs located within the -2 kb to 2 kb cis-regulatory regions of these genes.

### Statistical analyses

Methods used for statistical analysis of the microarray data are described in the results section. All other data were analyzed using ANOVA followed by Bonferroni post hoc comparisons (GraphPad Software, San Diego, CA) and significance was set at *P* ≤ 0.05.

## Results

### FoxO is necessary for locomotor and diaphragm wasting in C26 tumor-bearing mice

Wasting of locomotor muscles is an important component of whole body weakness and fatigue in cancer patients. In addition, the diaphragm muscle also undergoes significant wasting and is believed to play a key role in respiratory complications and mortality in cancer patients. Despite this, studies aimed at understanding the mechanisms of muscle wasting during cancer have largely focused on locomotor muscles. Thus, in the current study we focused on the role of the FoxO factors in cancer-induced wasting of both locomotor muscles and the diaphragm in response to Colon-26 tumor. To inhibit endogenous FoxO1, FoxO3a and FoxO4 DNA binding-dependent transcription we transduced muscles with recombinant AAV9-d.n.FoxO (or AAV9-ev as the respective control) both of which also express GFP as a non-fusion protein to visualize transduction efficiency. Importantly, the d.n.FoxO sequence consists of only that which codes for the FoxO3a DNA binding domain, and shares 85% sequence identity with the respective DNA binding domain of FoxO1 and 75% sequence identity with that of FoxO4 (Figure 1), all of which share >90% sequence conservation within this region. The d.n.FoxO therefore acts through outcompeting endogenous FoxO1, FoxO3a and FoxO4 for binding to FoxO DNA binding elements, and, since it lacks a transactivation domain, blocks FoxO DNA binding-dependent transcription. To transduce locomotor muscles we performed a single intramuscular injection of AAV9 into the TA and EDL muscles of mice. To transduce the diaphragm, in a separate cohort of animals we performed a single intrathoracic injection of AAV9. Immediately following AAV9 injections, mice assigned to the tumor-bearing groups were inoculated with C26 cells, and control mice injected with 1×PBS. Muscles from control and tumor-bearing mice were harvested at tumor end point (~26 days post C26-inoculation) when mice lose ~15% of their tumor-free body mass, which has been documented by us previously [9]. Using these methods, we were able to achieve nearly 100% AAV9 transduction efficiency of fibers in the TA muscle and the diaphragm and ~75% transduction efficiency of fibers in the EDL as visualized by GFP fluorescence in muscle cross-sections (Figure 1).

**Figure 2:**
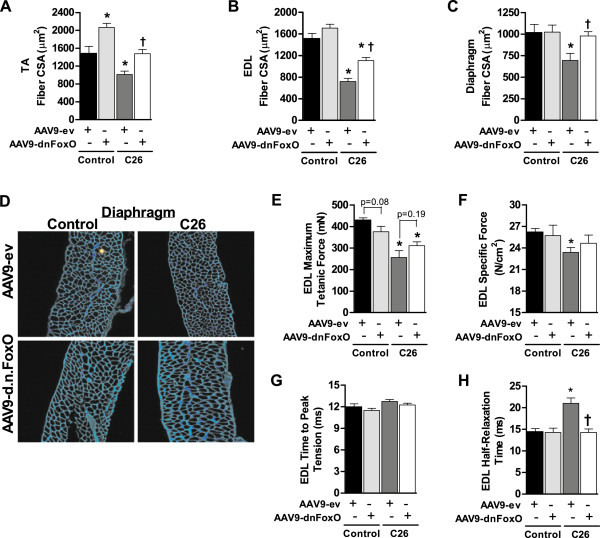
**Inhibition of FoxO impedes C26-induced muscle fiber atrophy and weakness. (A-D)** The average cross-sectional area of muscle fibers in the TA **(A)**, EDL **(B)** and diaphragm **(C)** of control or cachectic C26 mice transduced with AAV9-ev or AAV9-d.n.FoxO was calculated following incubation of muscle cross-sections with Alexa Fluor-conjugated wheat germ agglutinin to label muscle fiber membranes. **(D)** Representative diaphragm muscle cross-sections from each group. Data represent mean ± SE, n = at least 6 animals per group. **(E-H)** Maximum absolute tetanic force **(E)**, specific force **(F)**, time to peak tension **(G)** and half-relaxation time **(H)** was calculated in EDL muscles from control or cachectic C26 mice transduced with AAV9-ev or AAV9-d.n.FoxO. Data represent mean ± SE, n = 4 animals/group. *p < 0.05 vs AAV9-ev control group. †p < 0.05 vs. AAV9-ev C26 group.

As shown in Table 1 and Figure 2, presence of the C26 tumor induced significant muscle fiber atrophy in the TA, EDL, and diaphragm of mice transduced with AAV9-ev. In contrast, muscles of tumor-bearing mice transduced with AAV9-d.n.FoxO showed significant sparing of muscle fiber CSA. These data therefore demonstrate that blocking FoxO-dependent transcription is sufficient to impede C26-induced muscle wasting of both locomotor muscles and the diaphragm, which extends previous findings that FoxO is necessary for locomotor muscle wasting during LLC- and S-180-induced cancer cachexia [17]. Notably, transduction of muscles of non-tumor-bearing mice with AAV9-d.n.FoxO significantly increased fiber CSA over the 26 day period in the TA as observed previously by our lab. Increased fiber size was also observed in the EDL, but presumably due to the lower AAV9 transduction efficiency, this did not reach statistical significance (p = 0.16) since all fibers in muscle cross-sections were measured in order to make sense of subsequent muscle force measurements in the EDL. In contrast, fiber size was not altered by AAV9-d.n.FoxO in the diaphragm of control mice despite the high transfection efficiency. Although it is unclear why blocking FoxO induced hypertrophy of limb muscles, but not the diaphragm, this differential regulation may be related to the distinct activity pattern and function of the diaphragm, which is constantly active to support breathing.

**Table 1:** **Effect of AAV9-d.n.FoxO on muscle fiber CSA in C26 tumor-bearing mice**

	Control AAV9-ev	Control AAV9-d.n.Foxo	C26 AAV9-ev	C26 AAV9-d.n.FoxO
TA fiber CSA	1489 μm^2^ ± 155	2065^*^ μm^2^ ± 94	1013^*^ μm^2^ ± 76	1479^†^ μm^2^ ± 94
EDL fiber CSA	1518 μm^2^ ± 90	1705 μm^2^ ± 75	716^*^ μm^2^ ± 60	1106^*†^ μm^2^ ± 57
Diaphragm fiber CSA	1021 μm^2^ ± 91	1024 μm^2^ ± 82	696^*^ μm^2^ ± 81	980^†^ μm^2^ ± 50

*p < 0.05 vs control AAV9-ev group, †p < 0.05 vs. AAV9-ev C26 group. Data represent mean ± SE, n = 6 mice per group.

In order to determine whether the sparing of muscle fiber size in muscles of C26 tumor-bearing mice transduced with AAV9-d.n.FoxO carried over to sparing of muscle force deficits, we also harvested a subset of EDL muscles for measurement of *in vitro* contractile properties. The rationale for choosing the EDL (over the TA and diaphragm) for force measurements is due to two main reasons: 1) the relatively small size of the EDL allows for efficient diffusion of oxygen and nutrients necessary for force measurements (which is not possible in the TA), and; 2) the EDL contains tendons on both sides which allows for both specific and maximal absolute force measurements (maximal absolute force measurements are not possible in the diaphragm). We found that EDL muscles from C26 mice transduced with AAV9-ev showed a 40% decrease in maximum absolute force and an 11% decrease in specific force when compared to EDL muscles of control mice transduced with AAV9-ev, both of which were statistically significant (Figure 2E,F). In contrast, muscles from tumor-bearing mice transduced with AAV9-d.n.FoxO showed only a 28% decrease in maximum absolute force and a 6% (non-significant) decrease in specific muscle force, when compared to EDL muscles of control mice transduced with AAV9-ev. Although the attenuation of force deficits by AAV9-d.n.FoxO was not complete, these data are comparable with the effect of AAV9-d.n.FoxO on fiber size in EDL muscles of tumor-bearing mice, in which we saw only a partial sparing of fiber CSA due to the measurement of both transduced and non-transduced muscle fibers. Thus, given that only ~75% of fibers were transduced with AAV9-d.n.FoxO, it seems likely that a greater attenuation of muscle weakness would have occurred had we achieved a more complete transduction of fibers within the EDL. Notably, EDL muscles from control mice transduced with AAV9-d.n.FoxO over the 26 day period showed a non-significant (p = 0.09) decrease in maximum absolute muscle force when compared to AAV9-ev control, which suggests that chronically blocking FoxO in the absence of an atrophy stimulus may have a negative impact on force generating capacity.Further analysis of contractile properties of the EDL demonstrated no significant differences in time to peak tension in response to the C26 tumor or AAV9-d.n.FoxO (Figure 2G). In contrast, half-relaxation time was significantly slowed (elevated) in response to the C26 tumor, which was completely prevented in muscles transduced with AAV9-d.n.FoxO (Figure 2H). Collectively, these data indicate that FoxO-dependent transcription is necessary for C26-induced muscle atrophy of locomotor muscles and the diaphragm, and that FoxO activation is also causative in C26-induced muscle contractile dysfunction.

### Microarray analysis to identify direct or indirect FoxO target genes during C26 cancer cachexia

To comprehensively identify the gene networks changed in response to the C26 tumor which require FoxO-dependent transcription, we harvested TA muscles from control and cachectic C26 mice transduced with AAV9-ev or AAV9-d.n.FoxO for microarray analysis. We identified 2,194 genes that were differentially expressed between control and C26 mice injected with AAV9-ev (−1.5 ≥ fold change ≥ 1.5, q ≤ 0.01). Subsequent comparison of these genes between C26 mice injected with either AAV9-ev or AAV9-d.n.FoxO showed that 544 genes were differentially expressed in the presence of AAV9-d.n.FoxO (q ≤ 0.01and fold change ≥1.5). Out of these genes, 1 gene (Ip6k3) was significantly changed by AAV9-d.n.FoxO during control conditions (q < 0.01) and was thus eliminated as a downstream target of FoxO in response to the C26 tumor. Out of the remaining 543 genes regulated via a FoxO-dependent manner, 296 genes were upregulated in skeletal muscle due to the C26 tumor (see Additional file 1) and 247 were downregulated (see Additional file 2). To identify the broader gene networks, biological processes and canonical pathways regulated through FoxO in response to tumor burden, we functionally categorized these genes using the DAVID Bioinformatics database [34, 36] and the Broad Institute Molecular Signatures Database (MSigDB) [37]. Transcripts upregulated in response to the C26 tumor were analyzed separately from transcripts downregulated in response to the C26 tumor.

### Transcription factors, including Cebpb and AP-1, are downstream targets of FoxO in cachectic muscle

**Figure 3:**
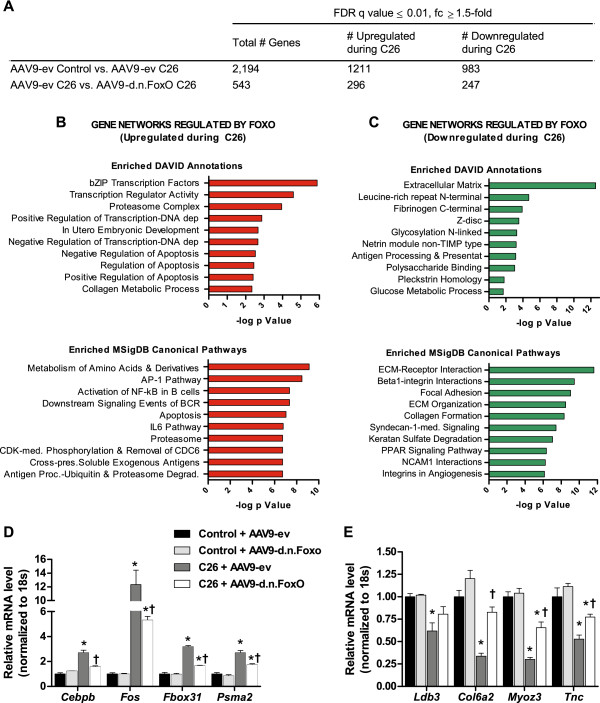
**Genome-wide identification of gene networks regulated by FoxO in muscles of cachectic C26 tumor-bearing mice.** **(** **A** **)** Microarray analyses were performed on TA muscles from control and cachectic C26 mice transduced with either AAV9-ev or AAV9-d.n.FoxO. Comparison of control and C26 AAV9-ev groups revealed 2,194 gene transcripts which were differentially expressed in response to C26 (FDR q-value < 0.01 and fold-change ≥ 1.5-fold). Of these genes, 543 genes were differentially regulated in muscles from C26 mice transduced with d.n.FoxO (AAV9-ev C26 vs. AAV9-d.n.FoxO C26, FDR q-value < 0.01 and fold-change ≥ 1.5-fold) and were thus considered as downstream targets (direct or indirect) of FoxO. **(** **B** **and** **C** **)** Gene transcripts upregulated **(** **B** **)** or downregulated **(** **C** **)** in response to C26 which were identified as FoxO targets were analyzed using the DAVID functional annotation clustering module and Broad’s Molecular Signature Database to identify enriched biological processes and Canonical Pathways. The top 10 most highly enriched DAVID annotation clusters and MSigDB Canonical Pathways from each gene set are ranked in order of significance and are plotted against the -log of the p-value. **(** **D** **and** **E** **)** Gene expression changes of select transcripts identified via microarray as downstream targets of FoxO were validated using qRT-PCR analyses. Data represent mean ± SE, n = at least 3 animals/group. *p < 0.05 vs AAV9-ev control group. †p < 0.05 vs. AAV9-ev C26 group.

Among the 296 direct or indirect FoxO target genes upregulated in skeletal muscle of C26 tumor-bearing mice, the most highly enriched biological annotation clusters identified through DAVID were related to the Basic Leucine Zipper (bZIP) transcription factors, the proteasome complex, transcriptional regulation and apoptosis. Ranked in order of significance, the most highly enriched annotation term from each of the top 10 non-redundant annotation clusters identified via DAVID are shown in Figure 3B (top panel). The top 10 Broad MSigDB canonical pathways, ranked in order of significance, are also shown in Figure 3B (bottom panel), which revealed findings consistent with the DAVID analysis. Among the top 20 canonical pathways, seven were related to protein degradation, including metabolism of amino acids, the proteasome, and antigen processing: ubiquitination and proteasome degradation. An additional seven pathways were associated with inflammatory processes, including AP-1, IL-6 and apoptosis. Expression data for FoxO-regulated transcripts belonging to enriched canonical pathways of interest are shown in Table 2.

**Table 2:** **Enriched gene networks upregulated via FoxO during cancer cachexia**

MSigDB pathway								C26 (Fold change)	
1	2	3	4	5	6	Gene description	Gene symbol	AAV9-ev	AAV9-dnFoxO*
x		x	x		x	proteasome subunit, alpha type, 7	Psma7	2.40	1.38
x		x	x		x	proteasome subunit, alpha type, 2	Psma2	2.95	1.73
x		x	x		x	proteasome 26S subunit, ATPase, 2	Psmc2	2.31	1.47
x		x	x		x	proteasome 26S subunit, ATPase, 4	Psmc4	2.47	1.49
x		x	x		x	proteasome 26S subunit, non-ATPase, 3	Psmd3	2.15	1.34
x		x	x		x	proteasome 26S subunit, non-ATPase, 4	Psmd4	2.67	1.69
	x					BCL2-like 11 (apoptosis facilitator)	Bcl2l11	3.01	1.82
	x			x		FBJ murine osteosarcoma oncogene	Fos	16.75	5.70
	x			x		jun B proto-oncogene	Junb	5.40	2.48
	x					early growth response 1	Egr1	4.07	1.95
	x					matrix metallopeptidase 9	Mmp9	2.52	1.57
	x					FOS-like antigen 2	Fosl2	3.50	2.09
	x					angiotensinogen	Agt	5.00	2.64
	x					FBJ murine osteosarcoma oncogene B	Fosb	3.90	1.43
		x			x	f-box and WD-40 domain protein 11	Fbxw11	1.88	1.15
			x	x		murine thymoma viral oncogene homolog 1	Akt1	2.61	1.54
			x	x		BCL2-like 1	Bcl2l1	3.01	1.82
				x	x	suppressor of cytokine signaling 3	Socs3	6.63	3.08
				x		CCAAT/enhancer binding protein beta	Cebpb	2.94	1.82
					x	kelch-like ECH-associated protein 1	Keap1	1.95	1.25
					x	splA/ryanodine receptor dom and SOCS box	Spsb1	6.36	3.91
***Other genes of interest***									
						immediate early response 5	Ier5	3.46	1.74
						immediate early response 3	Ier3	3.02	1.64
						ubiquilin-1	Ubqln1	1.93	1.25
						f-box protein 31	Fbxo31	3.25	1.79
						BCL2/adenovirus E1B interacting protein 3	Bnip3	2.53	1.51
						Cathepsin L	Ctsl	3.07	1.79
						GABA(A) receptor-associated protein like 1	Gabarapl1	4.44	2.52
						Heme Oxygenase 1	Hmox1	5.91	1.68

Expression changes of FoxO target genes of interest belonging to enriched Molecular Signatures Database (MSigDB) Canonical Pathways are shown for C26 tumor-bearing groups transduced with AAV9-ev or AAV9-d.n.FoxO.

All data represent fold-change in response to the C26 tumor, normalized to the absolute control group (AAV9-ev control). *q<0.01 vs AAV-ev C26 group. MSigDB Canonical Pathways: 1. Metabolism of Amino Acids and Derivatives, 2. AP-1, 3. Activation of NF-κB in B-cells, 4. Apoptosis, 5. IL-6, 6. Antigen Processing: Ubiquitination and Protein Degradation.

Among the FoxO-regulated genes annotated to the canonical IL-6 and/or AP-1 pathways are several bZIP transcription factors including CCAAT/enhancer-binding protein beta (*Cebpb)*, and factors which heterodimerize within the AP-1 transcription factor complex, including v-fos FBJ murine osteosarcoma viral oncogene homolog (*Fos*), *Fosl2*, *Fosb* and Jun B proto-oncogene (*Junb)*. Based on these findings we postulated that transcription factor binding motifs for C/EBPβ and AP-1, in addition to FoxO, would be enriched in the promoter regions of genes identified as indirect or direct targets of FoxO. Thus, in a separate analysis we used Broad’s Gene Set Enrichment Analysis tool to analyze the -2 kb to 2 kb cis-regulatory regions surrounding the transcriptional start site of *upregulated* FoxO target genes to identify the most commonly shared conserved transcription factor consensus motifs. This tool uses overlap comparison of user-provided gene lists and gene sets defined in the TRANSFAC (version 7.4) database as those which share a cis-regulatory motif conserved across the human, mouse, rat and dog genomes. Due to the conservation of these motifs across species, these sites are more likely to reflect putative gene regulatory elements. As expected, a TTGTTT consensus motif, which is annotated to FoxO4 (q = 3.76E-13), and is part of the core FoxO consensus motif also regulated by FoxO1 and FoxO3a, was identified as the second most commonly shared motif (see Additional file 3: Table S1). Moreover, although less commonly shared, the complete reverse FoxO consensus motif (G/A)TAAACA (annotated to FOXF2, q = 2.08E-06), which matches the FoxO DNA binding element (FBE) regulated by FoxO1 and FoxO3a in the MuRF1 promoter [38], was also significantly shared among the genes in our dataset. In addition, as hypothesized a motif corresponding to AP-1 (TGANTCA, q = 8.33E-12) was identified as the third most commonly shared motif. Thus, although associative, these findings at least support the notion that a subset of the genes identified as downstream targets of FoxO during cancer may be related to FoxO-dependent regulation of AP-1 transcription factors. Also among the top 10 most commonly shared consensus motifs were two motifs annotated to STAT5. Although the role of STAT5 in skeletal muscle wasting is unknown, STAT3 binds to an analogous motif and was recently shown to mediate muscle atrophy in C26 tumor-bearing mice [11]. STAT3 is activated through the IL-6 Pathway, which was also among the top 10 canonical pathways regulated by FoxO in response to tumor burden. Expression data for gene transcripts regulated by FoxO that are annotated to the IL-6 pathway are shown in Table 2, which includes the bona fide STAT3 target gene, suppressor of cytokine signaling 3 (*Socs3)*.

### Proteasome components enriched among FoxO targets upregulated in cachectic muscle

Based on the analyses performed using both DAVID and the MSigDB, genes involved in proteasomal protein degradation were enriched among the gene transcripts upregulated in cachectic muscle through a FoxO-dependent manner. Expression data for FoxO-regulated genes annotated to the “Proteasome” and “Antigen processing: ubiquitination and proteasome degradation” are shown in Table 2. Included are genes that encode for various subunits of the 26S proteasome, including the 20S core (*Psma2* and *Psma7*) and the 19S regulator (*Psmc2, Psmc4, Psmd3* and *Psmd4*) which confers substrate specificity to the 26S complex. Several additional genes which function as E3 ligases were also identified as downstream targets of FoxO, including F-box/WD repeat-containing protein 11 *(Fbx11), Socs3*, kelch-like ECH-associated protein 1 (*Keap1*), splA/ryanodine receptor domain and SOCS box containing 1 (*Spsb1)* and F-box protein 31 (*Fbxo31)* (Table 2)*.* In addition, Cathepsin-L, *Gabarapl1* and *Bnip3*, which are known gene targets of FoxO in skeletal muscle involved in protein degradation through the lysosomal/autophagy pathway [39, 40], were also identified as FoxO targets during cancer. Atrogin-1/MAFbx (*Fbxo32)* and MuRF1 *(Trim63),* although slightly repressed by d.n.FoxO, did not pass the statistical criteria set for the identification of FoxO-dependent targets in limb muscles of cachectic mice (see Additional file 3: Table S2). This finding therefore suggests that in addition to FoxO, other factors likely contribute to the transcriptional upregulation of atrogin-1 and MuRF1 during cancer cachexia, which has been reported previously [13]. Alternatively, since the d.n.FoxO construct inhibits FoxO-dependent transcription through outcompeting endogenous FoxO for DNA binding, it is also possible that endogenous FoxO factors could still regulate atrogin-1 and MuRF1 transcription in the presence of d.n.FoxO through a DNA-binding independent manner.

### FoxO is necessary for cancer-induced downregulation of genes encoding ECM and Z-disc proteins

In addition to the 296 FoxO target genes upregulated in skeletal muscle of C26 tumor-bearing mice, FoxO was also necessary for the C26-induced downregulation of 247 genes. Analysis of these genes using the Functional Clustering Tool within DAVID identified the Extracellular Matrix, Leucine-Rich Repeats, the Z-disc and Glycosylation among the most highly enriched annotations (Figure 3C, top panel). Use of the Broad Molecular Signatures Database to identify top canonical pathways from this gene set further revealed ECM-receptor interactions, β1-integrin interactions, focal adhesion, ECM organization and collagen formation as the top canonical pathways (Figure 3C, bottom panel). Expression changes of downregulated genes belonging to enriched annotation categories of interest are shown in Table 3. Several of these targets have previously been documented via microarray analysis to be downregulated in skeletal muscle of cachectic C26 tumor-bearing mice [9, 12], though this is the first evidence demonstrating the requirement of FoxO for their downregulation. Included among the genes downregulated in a FoxO-dependent manner were those encoding for Type I and Type VI collagens. As shown in Table 3, these collagens are part of several ECM-related pathways, and play an important role in maintaining the structural integrity of muscle during contraction. In addition, several small leucine-rich repeat proteoglycans (SLRPs) were also identified as FoxO-regulated transcripts downregulated in cachectic muscle, including Chondroadherin*,* Keratocan, Osteoglycin, Asporin, Fibromodulin and Lumican*.* While the functions of these SLRPs in skeletal muscle are not well understood, these proteins are located in the ECM where they regulate the structure and integrity of the ECM, and can also modulate growth factor interactions with their cellular receptors [41]. Also enriched among the downregulated transcripts regulated downstream of FoxO were several genes whose protein products localize to the muscle sarcomere, and in particular, the Z-disc (Table 3). Included among these are Cypher, also known as ZASP or Limb domain binding protein 3 (*Ldb3),* Homer1 and Myozenin 3 (*Myoz3*, also known as calsarcin-3), all of which play critical roles in muscle fiber integrity and function [42–44]. Although the significance of these ECM and Z-disc proteins during cancer cachexia is unknown, the integrity of the sarcomere as well as the muscle fiber membrane, which is tightly linked with the ECM, is notably disrupted in cachectic muscle [6, 45].

**Table 3:** **Enriched gene networks downregulated via FoxO during cancer cachexia**

MSigDB pathway							C26 (Fold change)	
1	2	3	4	5	Gene description	Gene symbol	AAV9-ev	AAV9-dnFoxO*
x	x	x	x	x	collagen, type I, alpha 2	Col1a2	−4.26	−2.31
x	x	x	x	x	collagen, type I, alpha 1	Col1a1	−5.20	−2.44
x	x	x	x	x	collagen, type VI, alpha 1	Col6a1	−2.64	−1.70
x	x	x	x	x	collagen, type VI, alpha 2	Col6a2	−2.69	−1.71
x	x	x	x	x	collagen, type VI, alpha 3	Col6a3	−3.55	−1.75
x	x	x			thrombospondin 2	Thbs2	−1.62	−1.07
x	x	x			tenascin C (hexabrachion)	Tnc	−2.43	−1.45
x		x			integrin, beta 6	Itgb6	−10.24	−2.20
x		x			cartilage oligomeric matrix protein	Comp	−2.48	−1.05
x		x			chondroadherin	Chad	−16.34	−2.40
	x				fibrillin 1	Fbn1	−2.86	−1.71
		x			Rho-associated, coiled-coil protein kinase2	Rock2	−1.98	−1.16
			x	x	collagen, type XIV, alpha 1 (undulin)	Col14a1	−2.08	−1.26
			x	x	procollagen C-endopeptidase enhancer	Pcolce	−1.81	−1.10
			x		matrix metallopeptidase 15	Mmp15	−3.12	−2.06
**DAVID annotation**								
**1**	**2**	**3**	**4**	**5**				
x	x	x			myozenin 3	Myoz3	−5.53	−1.99
x	x	x			integrin beta 1 binding protein 2 (melusin)	Itgb1bp2	−4.25	−2.07
x	x	x			junctophilin 2	Jph2	−3.95	−2.10
x	x	x			synemin, intermediate filament protein	Synm	−2.61	−1.47
x	x	x			homer homologue 1	Homer1	−2.26	−1.45
x	x	x			LIM domain binding 3/Zasp/Cypher	Ldb3	−1.84	−1.14
			x	x	fibromodulin	Fmod	−3.29	−1.29
			x	x	keratocan	Kera	−14.38	−3.30
			x	x	lumican	Lum	−2.47	−1.43

Expression changes of FoxO target genes belonging to enriched Molecular Signatures Database (MSigDB) Canonical Pathways and DAVID functional annotation categories of interest are shown for C26 tumor-bearing groups transduced with AAV9-ev or AAV9-d.n.FoxO.

All data represent fold-change in response to the C26 tumor, normalized to the absolute control group (AAV9-ev control). *q<0.01 vs AAV-ev C26 group. MSigDB Canonical Pathways: 1. Extracellular Matrix (ECM) Receptor Interactions, 2. Integrin1 Pathway, 3. Focal Adhesion, 4. ECM Organization, 5. Collagen Formation. DAVID Functional Annotations: 1. Z-disc, 2. I-band, 3. Sarcomere, 4. N-linked Glycosylation, 5. Small leucine-rich repeat proteoglycan.

In order to gain insight into the mechanisms whereby FoxO may contribute to gene downregulation during cancer cachexia we used the Broad Institute’s Gene Set Enrichment Analysis tool to identify common transcription factor consensus motifs located within the promoter regions of genes whose downregulation during cancer required FoxO. Among the top 10 most commonly shared conserved transcription factors consensus motifs were those corresponding to nuclear factor of activated T-cells (NFAT), FOXO4, myocyte-specific enhancer factor 2A (MEF2A), serum response factor (SRF), and myogenic differentiation 1 (MyoD) (see Additional file 3: Table S3).

### qRT-PCR validation of transcripts regulated by FoxO

To validate a subset of the transcripts identified via microarray as downstream targets of FoxO during C26, we performed qRT-PCR using cDNA generated from the same RNA samples used in the microarrays. As shown in Figures 3D and E, similar to our findings using microarray analysis, FoxO was necessary for the cancer-induced upregulation of *Cebpb*, *Fos*, *Fbxo31* and *Psma2*, and was necessary for the cancer-induced downregulation of *Col6a2*, *Myoz3* and *Tnc* (interaction effect, p < 0.05, AAV9-ev C26 vs. AAV9-d.n.FoxO C26, p < 0.05). In contrast, the levels of Cypher/*Ldb3* were not statistically different between the AAV9-ev C26 vs. AAV9-d.n.FoxO C26 groups.

### FoxO-dependent gene transcripts in the diaphragm during cancer cachexia

**Figure 4:**
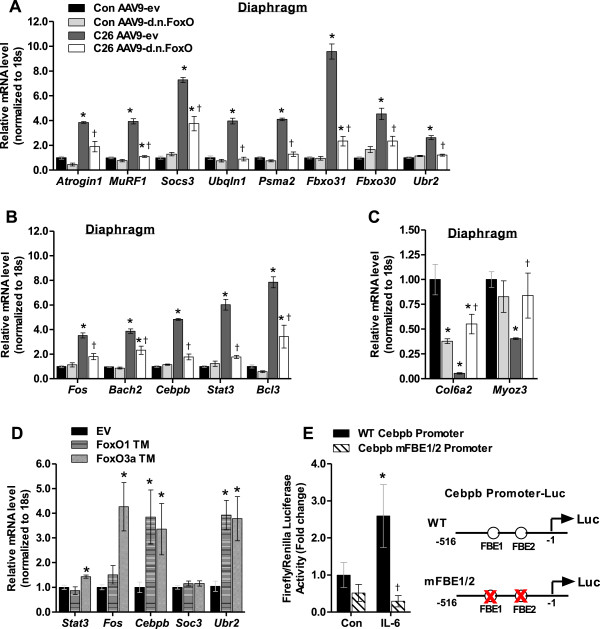
**FoxO1 and FoxO3a regulate** ***Cebpb*** **,** ***Fos*** **and** ***Stat3*** **gene expression. (A-C)** Select transcripts identified via microarray as downstream targets of FoxO in locomotor muscles during C26 cancer cachexia were validated as targets in the diaphragm via qRT-PCR analyses. Data represent mean ± SE, n = 3–5 animals/group. *p < 0.05 vs AAV9-ev control group. †p < 0.05 vs. AAV9-ev C26 group. **(D)** FoxO1 TM or FoxO3a TM expression plasmids were injected and electroporated into TA muscles of mice and harvested 4 days later for qRT-PCR analyses. Data represent mean ± SE, n = 5 animals/group. *p < 0.05 vs empty vector (EV). **(E)** C2C12 myoblasts were transfected with a pRL-TK-Renilla reporter plus a WT *Cebpb* promoter reporter construct or a *Cebpb* reporter construct which is mutated at two putative Forkhead binding elements, which prevents FoxO DNA binding. Following 3 days of differentiation, myotubes were treated with IL-6 (10 ng/mL) for 3 hours and harvested for measurement of firefly/renilla luciferase activity. Data represent mean ± SE, n = at least 9 wells/group. *p < 0.05 vs control. †p < 0.05 vs. WT Cebpb promoter.

As shown in Table 1, inhibition of FoxO-dependent transcription completely prevented diaphragm muscle fiber atrophy during C26-induced cachexia. Therefore, in order to determine whether FoxO regulates similar gene targets in the diaphragm as in the TA, we performed qRT-PCR analyses on diaphragm muscles. Included in these analyses were also transcripts of interest that marginally missed the fold-change and/or significance criteria to be classified as FoxO-dependent transcripts in response to cancer in the TA (see Additional file 3: Table S2). This included *Stat3* and the ubiquitin E3 ligases atrogin-1/MAFbx, MuRF1, *Ubr2* and *Fbxo30,* the latter of which was recently identified as a FoxO target gene necessary for denervation-induced atrophy [46]. As shown in Figure 4A, similar to the TA, diaphragm muscles from C26 mice showed significant increases in the gene transcripts of various genes involved in protein degradation, including *Socs3*, *Ubqln1, Psma2, Fbxo31, Fbxo30,* and *Ubr2*, all of which were significantly repressed in diaphragm muscles of C26 mice transduced with AAV9-d.n.FoxO. We also found, similar to the TA, numerous transcription factor transcripts to be upregulated in the diaphragm in response to C26, including the b-ZIP transcription factors, *Fos, Bach2* and *Cebpb,* as well as *Stat3* and *Bcl3* (Figure 4B), each of which were significantly repressed or abolished in diaphragm muscles transduced with AAV9-d.n.FoxO. We also found that similar to the TA muscle, in response to cancer diaphragm muscles showed a significant downregulation of the collagen transcript, *Col6a2*, and *Myoz3*, which encodes the Z-disc-associated protein Myozenin 3, and that their downregulation was blocked in muscles transduced with AAV9-d.n.FoxO (Figure 4C). In addition, the C26-induced increase in atrogin-1 (*Fbxo32)* and MuRF1 (*Trim63)* in the diaphragm were significantly repressed by d.n.FoxO. This finding is in contrast to the TA, where their repression by d.n.FoxO did not reach statistical significance. This discrepancy could be related to the increased sensitivity of qRT-PCR in comparison to microarray. We therefore validated the effect of d.n.FoxO on the C26-induced increase in atrogin-1 and MuRF1 in TA muscles via qRT-PCR, and found a greater magnitude of repression than indicated on the microarray (~40% repression of atrogin-1, ~60% repression of MuRF1), both of which reached statistical significance (p < 0.05, data not shown). However, since the magnitude of repression by d.n.FoxO was still considerably greater for both atrogin-1 and MuRF1 in the diaphragm than in the TA, these data suggest that additional transcription factors perhaps more active in limb muscle also play a role in atrogin-1 and MuRF1 transcription during cancer cachexia, which has been reported previously [13].

### FoxO1 and/or FoxO3a are sufficient to upregulate *Fos, Cebpb,*and *Stat3*

The identification of *Cebpb, Fos and Stat3* as downstream targets (direct or indirect) of the FoxO factors during cancer cachexia is significant, as each of these transcription factors have been identified to regulate the muscle atrophy program. More specifically, *Cebpb* is necessary for muscle wasting during LLC-induced cancer cachexia [13], *Stat3* for muscle wasting during C26 cancer cachexia [11], and *Fos* for denervation-induced muscle loss [47]. Moreover, the heterodimeric AP-1 transcription factor containing c-JUN, (which commonly dimerizes with FOS) is also necessary for muscle wasting in AH-130 tumor-bearing rats, which further implicates FOS in cancer-induced muscle wasting [14]. To further establish the FoxO factors as upstream regulators of these atrophy-related transcription factors, we next determined whether FoxO1 and/or FoxO3a were sufficient to increase the mRNA levels of *Cebpb*, *Fos* and *Stat3* in skeletal muscle. We also determined whether FoxO1 and FoxO3a were sufficient to increase the expression of *Socs3* and *Ubr2*, which are bona fide targets of STAT3 and C/EBPβ, respectively. We therefore injected and electroporated into skeletal muscle expression plasmids encoding “constitutively active” FoxO1 (FoxO1 TM) or FoxO3a (FoxO3a TM) which are mutated at the three Akt phosphorylation sites and harvested muscles 4 days later for qRT-PCR analysis. As shown in Figure 4D, FoxO1 TM significantly increased the mRNA levels of *Cebpb* (3.8-fold) and the C/EBPβ target gene, *Ubr2* (3.9-fold). On the other hand, FoxO3a TM significantly increased the mRNA levels of *Stat3* (1.4-fold), *Fos* (4.3-fold), *Cebpb* (3.1-fold) and *Ubr2* (3.8-fold), but not *Socs3*. Since both FoxO1 and FoxO3a were sufficient to increase *Cebpb* mRNA to similar magnitudes, we analyzed the *Cebpb* proximal promoter and identified two potential Forkhead boxO binding elements (FBEs) within 300 base pairs upstream of the transcription start site. A literature search revealed that FoxO1 binds to these sites within the *Cebpb* promoter to regulate *Cebpb* transcription in adipocytes in response to TNFα [25]. We therefore determined whether these FBEs within the *Cebpb* promoter are also necessary for increased *Cebpb* transcription in response to IL-6, a predominant cytokine in the C26 model of cancer cachexia. To test this we transfected C2C12 skeletal muscle cells with luciferase reporter plasmids driven by either a wild type fragment of the *Cebpb* proximal promoter (−516 to −1) or a mutated fragment (mFBE1/2) which contains base pair substitutions at both FBEs within the *Cebpb* promoter which prevent FoxO DNA binding [25]. Following differentiation into myotubes we treated cells with IL-6 for 3 hours and then harvested cells for measurement of luciferase activity. As shown in Figure 4E, treatment with IL-6 significantly increased *Cebpb* promoter reporter activity, which required the intact FBEs.

## Discussion

Muscle wasting during cancer is a unique and multifactorial process which likely involves the differential regulation of multiple signaling pathways and downstream targets that together promote the wasting phenotype. However, the specific muscle proteins which mediate these intracellular responses to tumor burden are only beginning to be defined. In the current study we extend recent findings that the FoxO transcription factors are necessary for locomotor muscle fiber atrophy during Lewis Lung carcinoma [16] and S-180 sarcoma [17] to show that FoxO is required for skeletal muscle fiber atrophy in response to C26 adenocarcinoma in both locomotor muscles and the diaphragm, the primary muscle required for inspiration. This is significant, since diaphragm wasting during cancer may link the degree of cachexia with increased mortality. Importantly, through performing the first genome-wide microarray analysis of transcripts regulated in a FoxO-dependent manner in skeletal muscle during cancer cachexia, we reveal several gene networks changed in cachectic muscle that require FoxO. Indeed, while the FoxO factors are well known to regulate genes involved in protein degradation which was confirmed through our study, we provide new evidence that during cancer FoxO is also necessary for the gene upregulation of various atrophy-related transcription factors (and their associated transcriptional networks), including Cebpb, Stat3 and AP-1. In addition, we further demonstrate that FoxO also plays a key role in mediating the cancer-induced downregulation of various genes which function in the maintenance of muscle structural integrity. Collectively these findings highlight FoxO as a critical factor controlling diverse transcriptional networks in skeletal muscle during cancer cachexia, which provides novel insight into additional mechanisms whereby the FoxO transcription factors may orchestrate the muscle atrophy phenotype.

### Atrophy-related transcription factors identified as downstream targets of FoxO in response to tumor burden

Several transcription factors were identified through microarray analysis as downstream targets of FoxO in response to tumor-burden, including the atrophy-related bZIP transcription factors, *Cebpb* and *Fos*. The identification of *Cebpb* as a downstream target of FoxO in skeletal muscle during cancer is of particular interest, since the protein expression of C/EBPβ is increased in muscles of tumor bearing mice, and mice lacking C/EBPβ are resistant to LLC cancer-induced muscle wasting [13]. Moreover, we also found that both FoxO1 and FoxO3a are sufficient to increase *Cebpb* mRNA in skeletal muscle. Although we cannot ascertain, based on our findings, whether FoxO1 or FoxO3a *directly* induce *Cebpb* gene transcription during cancer cachexia, there are two putative FBEs located within the *Cebpb* proximal promoter that FoxO1 has previously been documented to bind [25]. Here we show that these FBEs are necessary for *Cebpb* promoter activation in skeletal muscle in response to IL-6, a predominant cytokine in the C26 model of cancer cachexia. Given the requirement of C/EBPβ for cancer-induced muscle wasting, it seems likely that FoxO-dependent upregulation of *Cebpb* plays a role in the muscle wasting phenotype induced by FoxO during cancer.

The significance of the immediate early gene and oncogenic transcription factor, FOS, as a FoxO target in skeletal muscle during cancer cachexia is currently unknown. However, *Fos* was the third most highly upregulated FoxO target gene in response to C26 (16-fold), which is in alignment with a previous microarray study showing upregulation of *Fos* in muscles from both moderately and severely cachectic C26 tumor-bearing mice [12]. Importantly, *Fos* is also upregulated in skeletal muscle following denervation and knockdown of *Fos* prevents the associated muscle atrophy, thus highlighting its role in the atrophy program [47]. Despite the unknown role of FOS in cancer-induced wasting, FOS heterodimerizes with c-JUN within the AP-1 transcription factor complex, which has been established as a factor required for muscle wasting in AH-130 tumor-bearing rats [14]. Since the AP-1 pathway was identified as a top canonical pathway regulated by FoxO in cachectic muscle, it seems logical that FoxO-dependent induction of *Fos* could play a role in the enrichment of this pathway. Notably, in addition to *Fos* we also found that FoxO was necessary for the C26-induced increase in other early response genes related to the AP-1 pathway, including immediate early response 5 (*Ier5),* and early growth response 1 (*Egr-1*). Importantly, both *Egr-1* and *Ier5* were recently identified as cachexia-associated genes upregulated in muscles of pancreatic cancer patients together with *Foxo1*[18]. Erg-1 was increased greater than 60-fold in patients exhibiting cachexia compared to non-cachectic cancer patients, and is considered a master regulator of inflammatory response. To our knowledge these findings are the first to link FoxO to the regulation the AP-1 pathway during cancer cachexia which represents a potentially novel role for FoxO in mediating the wasting phenotype during cancer.

### FoxO regulates proteolytic genes in skeletal muscle in response to tumor burden

Many of the upregulated genes identified as downstream targets of FoxO in muscles of C26 tumor-bearing mice are involved in protein degradation, and are coordinately upregulated during multiple wasting conditions [48–50]. Included among these were various proteasome subunits and Ubiquilin-1 (*Ubqln1)* which encodes for an ubiquitin-like (UBL) protein which physically interacts with both proteasomes and ubiquitin ligases and is involved in protein degradation [51]. In addition, several genes whose protein products function in ubiquitin E3 ligase complexes were also identified as downstream targets of FoxO, including *Socs3, Fbxw11, Keap1*, *Spsb1* and *Fbxo31*. While little is known about the substrates and functions of these E3 ligases in skeletal muscle, Fbxw11 (also known as β-TrCP2) plays a role in targeting IκBα for ubiquitin-dependent degradation [52]. This finding is intriguing, since the degradation of IκBα is necessary for muscle wasting during cancer [7, 9, 10]. Importantly, FoxO is well-established to regulate proteolysis during wasting conditions [39, 40, 53], and we confirmed several known FoxO targets involved in proteolysis as dependent targets of FoxO during cancer. Included among these were autophagy-related target genes (*Bnip3*, Cathepsin-L and *Gabarapl1*), as well as *Fbxo30/*MUSA1, which was recently reported as a novel FoxO target gene which encodes an ubiquitin E3 ligase that is required for denervation-induced muscle loss [46]. Thus, based on these findings, it is reasonable to speculate that FoxO-dependent upregulation of these genes involved in proteolysis through both the ubiquitin proteasome pathway and the lysosomal/autophagy pathway likely plays an important role in cancer-induced wasting.

### FoxO is necessary for ECM and sarcomere gene downregulation in response to tumor burden

Unexpectedly, nearly half of the genes regulated by FoxO during cancer were downregulated in response to the C26 tumor. While FoxO could mediate gene repression through direct binding to gene promoters, as has been documented previously [54–56], FoxO may also regulate gene repression through indirect mechanisms. This is suggested by the promoter analyses performed on downregulated target genes which identified enrichment of conserved binding motifs for not only FoxO, but several transcription factors that regulate muscle gene products, including NFAT, MEF2, and MyoD. Thus, FoxO could indirectly contribute to gene repression through inhibiting the activity of these and other transcription factors. One mechanism whereby FoxO could mediate this is through increasing the expression of transcriptional repressors and co-repressors. In fact, inhibitor of DNA-binding/differentiation proteins 1 *(Id1*) and 3 (*Id3),* which act as transcriptional repressors of myogenic basic helix-loop-helix (bHLH) proteins such as MyoD [57], were both identified as downstream targets of FoxO increased in cachectic muscle. The direct and indirect mechanisms whereby FoxO contributes to gene repression during cancer thus warrant further investigation, and will be important in moving this work forward.

A significant number of the downregulated FoxO targets during cancer encode for proteins that localize to the ECM and the Z-disc of the muscle sarcomere. Despite the relatively long half-life of typical structural proteins, prolonged downregulation of these transcripts over several weeks or even months during the progression of cancer should logically be sufficient to decrease protein expression. Provided this holds true for even a small subset of the downregulated targets, the impact of their downregulation could be highly significant to the progression of cancer-induced wasting and weakness. Indeed, many of the target genes downregulated via FoxO play critical roles in maintaining muscle integrity, fiber size and contractile function. Just one example, the extracellular matrix protein, Tenascin C (Tnc), is increased in response to muscle damage, exerts anabolic and proliferative effects on interstitial and myogenic cells, and intriguingly, its genetic ablation is sufficient to decrease muscle mass, cause selective atrophy of type II muscle fibers, and slow muscle contractile properties [58]. Since these phenotypes are also characteristic of muscle wasting during cancer, it is intriguing to speculate that the downregulation of Tnc could play a role in these pathologies.

Strikingly, many of the ECM-related FoxO target genes downregulated in cachectic muscle of C26 tumor-bearing mice, including the collagen VI encoding genes, are predominately expressed and secreted by interstitial fibroblasts [59]. Indeed, fibroblasts and other stromal cells are the primary cells responsible for synthesizing the ECM and the collagen network. Although the effect of cancer on the muscle ECM and collagen network are not well defined, fibroblast activation protein-α (FAP)-positive stromal cells are significantly reduced in skeletal muscles of C26 tumor-bearing mice and depletion of these cells is sufficient to induce muscle fiber atrophy [60]. Moreover, collagen VI produced from muscle fibroblasts is critical to the structural integrity and function of the muscle and is also an important component of the satellite cell niche, that is necessary for satellite cell self-renewal and muscle regeneration [61]. In addition to repressing the expression of several collagen transcripts, we also found that FoxO was necessary for the coordinate increase in the gene expression of matrix metalloproteinase 8 (*Mmp8*) and *Mmp9* in muscles of cachectic tumor-bearing mice (Table 2 and Additional file 3: Table S1), which participate in the degradation of Type I, II and III collagens and Type IV collagens, respectively. Therefore, determining whether the ECM and collagen network are disrupted in skeletal muscle during cancer cachexia, and whether this is mediated through a FoxO-dependent manner, warrants further investigation. Moreover, since we know that fibroblasts are the predominant source of collagen and other ECM components identified as downstream targets of FoxO during cancer, determining the role of the FoxO factors in regulating muscle fibroblasts as it relates to cancer cachexia also warrants further study.

## Conclusions

In summary, the current study identifies FoxO as a critical factor required for muscle wasting in locomotor muscles and the diaphragm in response to C26 cancer cachexia. Moreover, these findings provide new evidence that FoxO-dependent transcription is a central node controlling diverse gene networks in skeletal muscle during cancer cachexia which may act coordinately to regulate the atrophy phenotype. Indeed, our findings indicate that FoxO regulates not only genes involved in proteolysis, but also acts as an upstream regulator of several transcriptional networks, including C/EBPβ and the AP-1 and IL-6 pathways. Intriguingly, our findings also indicate that FoxO plays a critical role in the cancer-induced downregulation of genes involved in ECM and sarcomere structure, which suggests an entirely novel role for FoxO in regulating muscle integrity during cancer. The data presented in this study thus highlights several novel candidate genes and biological networks that are targeted by FoxO that may be further explored as causative factors in cancer-induced muscle wasting.

## Electronic supplementary material

Additional file 1: **List of FoxO target genes upregulated in skeletal muscle during C26 cancer cachexia.** (PDF 52 KB)

Additional file 2: **List of FoxO target genes downregulated in skeletal muscle during C26 cancer cachexia.** (PDF 189 KB)

Additional file 3: **Tables S1**, **S2 and S3.** Enriched transcription factor binding motifs in promoter regions of FoxO target genes upregulated **(Table S1)** and downregulated **(Table S3)** during C26 cancer cachexia. **Table S2.** Expression data for additional gene transcripts of interest. (PDF 90 KB)

## Abbreviations

AAV9: Adeno-associated virus serotype 9
bZIP: Basic Leucine Zipper
C26: Colon 26
C/EBPβ: CCAAT/enhancer-binding protein beta
CSA: Cross-sectional area
DAVID: Database for Annotation, Visualization and Integrated Discovery
d.n.: Dominant negative
ERK 1/2: Extracellular regulated kinase 1 and 2
FoxO: Forkhead box O
GFP: Green fluorescent protein
IRES: Internal ribosome entry site
LLC: Lewis lung carcinoma
MSigDB: Molecular Signatures Database
NF-κB: nuclear factor kappa B
p38 MAPK: p38 mitogen-activated protein kinase
TA: Tibialis anterior
WT: Wild type.
