# Supplementary material for: Genome-wide identification of FoxO-dependent gene networks in skeletal muscle during C26 cancer cachexia
Source: BMC Cancer. 2014 Dec 24;14:997. doi: 10.1186/1471-2407-14-997 (PMC4391468; doi:10.1186/1471-2407-14-997)
Supplement: Supplementary file 1 — Additional file 1: List of FoxO target genes upregulated in skeletal muscle during C26 cancer cachexia. (PDF 52 KB) [file 12885_2014_5246_MOESM1_ESM.pdf]

JUDGE et al.

SUPPLEMENTARY FILE 1

FoxO target genes upregulated in skeletal muscle of C26 tumor-bearing mice

Fold change (fc) in response to C26

fc ≥ 5.0	5 > fc ≥ 3.5	3.5 > fc ≥ 3.0	3.0 > fc ≥ 2.5	2.5 > fc ≥ 2.0		2.0 > fc ≥ 1.5	
Gm10309	Hamp2	Ier5	Musk	Bach2	Cirbp	Sidt2	Tmem189
Lcn2	Tgif1	Lrrc58	Cth	Nploc4	Heatr7a	Tceanc	Myo1e
Fos	Itpkc	Ddi2	Pi4k2a	Psmc4	Gsr	Sec23ip	Atf6
Slc39a14	Bex1	Fah	Gpr157	Catsper2	Tbc1d25	Nudt16	Zfp715
Ampd3	Ctxn3	Rassf4	Psma7	Alas2	Foxp1	Gm1614	Mtmr4
Maff	Tmbim1	Id1	Mir29a	Pex12	Adck4	1110012L19Rik	Snap29
Mmp8	Slc25a33	Rhou	Cebpb	Bsdc1	Fam63a	Brms1	Mrgpre
Acss1	Hgs	Snai3	Rasa3	Zfand2a	Id3	Tekt1	Slc29a1
Otud1	Gpnmb	Ddx56	Slc20a1	Por	Tle1	Mcoln2	Acox2
Nr4a3	Igfbp3	Pnpla7	Tsr1	Daxx	Prosc	Pcid2	Rnf185
Retnlg	Gabarapl1	Fbxo31	Fkbpl	Slc2a1	Lhpp	Ccdc86	Osbp2
Slc10a6	Slc15a4	Krt80	Slc43a2	Zrsr1	Bin3	Ivns1abp	C030046E11Rik
Btg2	Tcp11l2	Pir	Arhgef37	Atp6v1h	Nub1	Gtf3c4	Sephs2
Ifi30	Slc7a8	Zfp346	Rhbdf2	Psma2	Psmd3	Keap1	Btbd16
Ankrd1	Gclm	Fam55c	Mpzl2	Nufip1	Zfp771	Mta2	Itpk1
Sesn1	Mocs1	1110038D17Rik	Slc1a1	Aspscr1	Chka	Nat9	Mapk6
Irs2	Wdr62	Sdc4	Gaa	Zfp593	Fam3a	Spata24	Slc40a1
Socs3	Acot1	Cpxm1	Usp3	Panx1	Hsf4	Dnajb2	Gm6194
Spsb1	Apold1	Kng2	Igf1r	Abhd4	Pias4	Rusc2	Ccno
Doc2b	Egr1	Sim2	Anapc16	1810031K1	Fam46c	Prpf6	
Arid5a	Selp	100502885	Ern1	Ubxn4	Fiz1	Ubqln1	
Ngp	Cry2	Gadd45b	Tmem87b	Cdkal1	Nr4a2	Dync1li1	
Il6ra	Bcl2l1	Ctps2	Acvr1b	Gm12592	Rit1	Klhl22	
Hmox1	Mocos	Bcl3	Serpina3c	Als2	Rnd1	Pes1	
Sik1	Egln3	Abhd6	Stc1	Ubxn8	Adamts2	Stk11ip	
Serpina3m	Fosb	Rgs2	Trmt1	Slc7a5	Spry4	Dscr3	
Saa3	Mc5r	Ctsl	Pim1	Tbc1d17	Naglu	Slc25a44	
Ifitm6	Trib1	Itih4	Sat1	Psmc2	Armcx5	Fbxw11	
Arl4d	Sbno2	Zfp259	Mecp2	Osgin1	Thumpd3	Sh3bp2	
Junb	Klf15	Ccbl2	Adamts1	Tbx1	Rnf113a2	Cndp2	
Ppp1r15a	Aacs	Naa25	Pnmt	Dnttip2	2410003K1	Pgd	
Rasd1	Vdr	Wdr81	Psmd4	Sdad1	Elac2	Nsmce1	
Cnksr1	Adamts4	Ier3	2310028H24Rik	Ddx27	Kat5	Dpyd	
Gm129	Zwint	Bcl2l11	Ankrd33b	Dmxl2		1700019E19Rik	
Csrnp1	Setdb2		Clca1	Slc25a34		Cobll1	
Kcnk5	Ranbp9		Wbscr27	Sbds		Papd7	
D230025D16Rik	Plk3		Akt1	Tex264		Ftsjd2	
Mafk	Tbc1d15		E2f8	Aph1c		Wipi2	
Agt	Fosl2		Gm16516	Map3k14		Trmt61a	
			AI317395	Mtmr14		Mms19	
			Bnip3	Zscan21		Tmcc3	
			Mmp9	Chpf2		Lemd2	
			Ezh1	Slc25a38		Dusp26	
			Tut1	Lrrc32		Aldh8a1	
