# Supplementary material for: Genome-wide identification of FoxO-dependent gene networks in skeletal muscle during C26 cancer cachexia
Source: BMC Cancer. 2014 Dec 24;14:997. doi: 10.1186/1471-2407-14-997 (PMC4391468; doi:10.1186/1471-2407-14-997)
Supplement: Supplementary file 2 — Additional file 2: List of FoxO target genes downregulated in skeletal muscle during C26 cancer cachexia. (PDF 189 KB) [file 12885_2014_5246_MOESM2_ESM.pdf]

SUPPLEMENTARY FILE 2

FoxO target genes downregulated in skeletal muscle of C26 tumor-bearing mice

Fold change (fc) in response to C26

fc ≤ -5.0	-5.0 < fc ≤ -3.5	-3.5 < fc ≤ -2.8	-2.8 < fc ≤ -2.0		-2.0 < fc ≤ -1.5
Plcd4	Trim7	Smtnl2	Pgm2	Zfp759	Rock2
Chad	Abcd2	Pdp1	H2-Aa	Synpo2l	H2-Eb1
Kera	Prkag3	Osbpl6	Hspb3	Zfp454	Inpp4b
Aqp4	Atp1b4	Angpt1	Gatsl2	9530009M10Rik	Hspb2
Tfrc	Retnla	Stac3	Rhbdl1	Prkg1	Igsf10
Itgb6	Mir181b-2	Crhr2	Nqo2	Isoc1	Sbk2
Itgb1bp3	Angptl2	Dpp4	2310042D19Rik	Sorl1	Fcrls
Tnmd	Mir133a-2	9330159F19Rik	Cc2d2a	Pex11a	Naa50
Agbl1	Gdap1	Sfrp2	Apol6	Itgbl1	Sntb1
A530098C11Rik	Nnat	P2ry1	Col6a2	Penk	Dynlt3
Kcng4	Ifi203	Ecm2	Zfp72	Homer1	Angptl4
Mir133a-1	Col1a2	Fmod	Eepd1	Asph	Gm5595
Igh-6	Relt	Rxrg	Slc38a3	Ormdl1	Ldb3
BC023105	Itgb1bp2	Sobp	Raver2	Abca8a	Rcn3
Sypl2	Best3	Wnt5a	Arhgef9	Zfp799	Pcolce
Lrrc38	Npr3	Lrp2bp	Col6a1	Cacna2d1	Zfp595
Mettl11b	Itm2a	Nexn	Aimp2	Mcpt4	Nudt7
Pde4a	Gpd1	Kctd15	Synm	Msrb3	Actr3b
6430571L13Rik	Stxbp4	Mmp15	Coq7	Glb1l2	100504541
Frzb	Dusp10	100504404	Plekhh3	100504422	2310046A06Rik
Igfbp5	Jph2	2310010M20Rik	Casq2	Trim13	Cilp
Cyp4f39	Grb14	Nt5c1a	Pde7b	Pgam2	Xirp2
Ogn	Dynll2	Reep1	Ear2	Ccdc3	Tmem47
Myoz3	Slc4a4	Psd3	Calm3	Apobec2	Sim1
Mfap4	Fndc1	Tgfb2	Gm11710	Pecr	1700025G04Rik
Fam78a	Aspn	Prss23	100504147	Fkbp3	Tbc1d1
Col1a1	Akap6	Myom3	Abi3bp	Trdn	Pqlc3
Wfdc1	Hdac9	Ociad2	Fn3k	Serpinf1	Fabp4
Map2k6	Efhd1	Rab3a	Comp	Btbd6	Thbs2
Sema6c	Rasgrp3	Kcnn2	Pon3	Pltp	Nova1
Aqp7	Myoc	Sfrp4	Lum	Nr1d1	Cpt1a
	ORF63	Postn	Dhrs7c	Hs3st5	Srpk2
	Tmem25	Ppargc1b	9530002K18Rik	Sgcd	
	Slc8a3	Sema3c	Cilp2	Chodl	
	Filip1l	Cd74	Scn1b	Phtf2	
	Islr	Gm889	Ampd1	Col14a1	
	Atp1b2	Nkain1	Irf5	Olfr558	
	Sh3bgr	Ramp1	Tnc	Abca9	
	Itih5	Gpam	St8sia5	Wif1	
	Ache	Fbn1	Pptc7	Tm6sf1	
	Carns1	Tppp3	Atxn1	Lgals1	
	Asb18	Eml1	Arhgap20	Dapk2	
	Gsta4	1700020C11Rik	Palld	Fxyd6	
	Col6a3	Otub2	Akr1b10	Cyb5r1	
		Ces3	H2-Ab1	Anxa5	
			Fsd2		
			Nav2		
			Hr		
			Phkg1		
			Sorbs2		
