# Supplementary material for: Genome-wide identification of FoxO-dependent gene networks in skeletal muscle during C26 cancer cachexia
Source: BMC Cancer. 2014 Dec 24;14:997. doi: 10.1186/1471-2407-14-997 (PMC4391468; doi:10.1186/1471-2407-14-997)
Supplement: Supplementary file 3 — Additional file 3: Tables S1, S2 and S3. Enriched transcription factor binding motifs in promoter regions of FoxO target genes upregulated (Table S1) and downregulated (Table S3) during C26 cancer cachexia. Table S2. Expression data for additional gene transcripts of interest. (PDF 90 KB) [file 12885_2014_5246_MOESM3_ESM.pdf]

SUPPLEMENTARY TABLES S1 –S3

Table S1. Most commonly shared conserved transcription factor consensus motifs located in-2kb to 2kb cis-regulatory regions of FoxO target genes upregulated in skeletal muscle of C26 tumor-bearing mice.

Rank	Transcription Factor	Consensus Motif	K	k	k/K	q-value
1	SP1	GGGCGGR	2940	55	0.0187	0.00E+00
2	FoxO4[a]	TTGTTT	2061	44	0.0213	3.76E-13
3	AP1 (Jun)	TGANTCA	1121	31	0.0277	8.33E-12
4	TCF3	CAGGTG	2485	44	0.0177	1.23E-10
5	AP4	CAGCTG	1524	33	0.0217	5.69E-10
6	E4F1	GTGACGY	658	21	0.0319	4.97E-09
7	BACH2	SRTGAGTCANC	271	14	0.0517	1.73E-08
8	STAT5B[b]	TTCYNRGAA	335	15	0.0448	2.46E-08
9	p65	GGGRATTTCC	237	13	0.0549	2.91E-08
10	STAT5A[b]	NAWTTCYNGGAANYN	251	13	0.0518	5.29E-08
Additional motifs of interest						
26	FOXF2[c]	RTAAACA	919	20	0.0218	2.08E-06
89	FOXO3a	TNNTTGTTTACNTW	245	7	0.0286	2.15E-03

K = total number of genes in the TRANSFAC-defined gene set annotated to each motif.
k = total number of FoxO target genes overlapping the defined gene set.
q-value = false discovery rate (FDR) analogue of the p-value representing the overlay significance.
[a]Motif is part of core FoxO consensus motif recognized by FoxO1, 3 and 4.
[b]Motif matches consensus motif recognized by STAT3.
[c]Motif matches the FoxO binding element in the MuRF1 promoter.

Table S2. Gene expression changes of select genes of interest in skeletal muscle from control and cachectic C26 tumor-bearing mice transduced with AAV9-ev or AAV9-d.n.FoxO.

**Gene Symbol**	**Gene Description**	**C26 (fold change)**	
		**AAV9-ev**	**AAV9-d.n.FoxO**
Foxo1	forkhead box O1 transcription factor	2.70*	2.20
Foxo3	forkhead box O3 transcription factor	2.54*	2.05
Foxo4	forkhead Box O4 transcription factor	1.08	1.12
Fbxo32	f-box protein 32/atrogin-1/MAFbx	2.66*	2.27
Trim63	tripartite motif-containing 63/MuRF1	4.51*	3.70
Fbxo30	f-box protein 30/MUSA1	1.84[§]	1.27
Eif4ebp1	eukaryotic translation initiation factor 4E binding protein 1	3.50*	2.64
Stat3	signal transducer and activator of transcription 3/acute phase response factor	2.74*	1.95
Atf4	activating transcription factor 4	1.95*	1.53
Bcl3	B-cell CLL/lymphoma 3	3.08*	1.88[†]
Il6ra	IL-6 receptor alpha	5.94*	3.33[†]
Id1	inhibitor of DNA binding/differentiation 1	3.29*	1.73[†]
Id3	inhibitor of DNA binding/differentiation 3	2.17*	1.25[†]
Ky	kyphoscoliosis peptidase	-19.89[§]	-4.44[†]

All data represent fold-change in response to C26 normalized to the absolute control group (AAV9-ev control).
*****Significantly changed in response to C26 (control AAV9-ev vs C26 AAV9-ev, q<0.01, fold change ≥1.5-fold).
[§]Significantly changed in response to C26 (control AAV9-ev vs C26 AAV9-ev, q<0.05, -1.5≤fold change≥ 1.5-fold).
†Significantly changed by d.n.FoxO (C26 AAV9-ev vs C26 AAV9-d.n.FoxO, q<0.01, fold change ≥1.5-fold).

Table S3. Most commonly shared conserved transcription factor consensus motifs located in-2kb to 2kb cis-regulatory regions of FoxO target genes downregulated in skeletal muscle of C26 tumor-bearing mice.

Rank	Transcription Factor	Consensus Motif	K	k	k/K	q-value
1	NFATC	TGGAAA	1896	42	0.0222	0.00E+00
2	TATA	TATAAA	1296	35	0.027	0.00E+00
3	FOXO4	TTGTTT	2061	39	0.0189	2.73E-13
4	MEF2A	YTATTTTNR	697	24	0.0344	3.76E-13
5	SRF	GNCCAWATAWGGMN	241	16	0.0664	7.78E-13
6	E12/E47	CAGGTG	2485	40	0.0161	1.11E-11
7	unknown	NNNNNWKCTAWAAATAGMNNNN	238	14	0.0588	1.50E-10
8	MEF2A	ANKCTAWAAATAGMHNN	214	13	0.0607	5.03E-10
9	MYOD	GCANCTGNY	924	23	0.0249	5.03E-10
10	REPIN1	CAGCTG	1524	29	0.019	5.03E-10

K = total number of genes in the TRANSFAC-defined gene set annotated to each motif.
k = total number of FoxO target genes overlapping the defined gene set.
q-value = false discovery rate (FDR) analogue of the p-value representing the overlay significance.
